# Pneumonia, pleurisy, mediastinitis, and mediastinal cyst infection secondary to endobronchial ultrasound-guided transbronchial needle aspiration: A case report

**DOI:** 10.1097/MD.0000000000025973

**Published:** 2021-05-21

**Authors:** Wei Liu, Yongxue Wang, Weidong Zhang, Huaiqiu Wu, Zhiguang Liu

**Affiliations:** a Department of Pulmonary and Critical Care Medicine, Hunan Provincial People's Hospital, The First Affiliated Hospital of Hunan Normal University, Changsha; b Department of Pulmonary and Critical Care Medicine, The First People's Hospital of Yueyang, Yueyang, Hunan, P.R. China.

**Keywords:** complication, endobronchial ultrasound-guided transbronchial needle aspiration, mediastinal cyst

## Abstract

**Introduction:**

Endobronchial ultrasound-guided transbronchial needle aspiration (EBUS-TBNA) is less commonly used in nonmalignant diseases. In particular, its application in mediastinal cystic lesions has been reported less frequently. EBUS-TBNA is a reassuringly safe procedure with an overall complication rate less than 2%, and serious adverse event rate of 0.14% to 0.16%. The most common complications are infections (mediastinal cyst infection most seen).

**Patient concerns:**

A 28-year-old male presented to the hospital with mediastinal cyst that was incidentally discovered by computed tomography. There was no past history of the patient reviewed.

**Diagnosis:**

The cyst was identified as a round, anechoic structure by EBUS and serous fluid was aspirated. The carcino-embryonic antigen, mycobacterium tuberculosis DNA and cultures in the fluid were negative. Cytology analysis showed lots of lymphocytes and no malignant cells. The diagnosis of lymphangioma was confirmed based on the computed tomography and EBUS presentation, the nature of the aspirated fluid and the large number of mature lymphocytes within the cystic fluid.

**Interventions:**

Twenty-six hours after EBUS-TBNA, the patient complained of a fever with the highest temperature of 39°C, accompanied by a right-side chest pain, no other symptoms of were reported. The following examinations confirmed the diagnosis of pneumonia, pleurisy, mediastinitis and mediastinal cyst infection, while cultures from cyst and right pleural effusion were both negative. The patient was treated with Teicoplanin+Imipenem/cilastatin, and ultrasound guided transcutaneous catheterization drainage of mediastinal cyst and pleural effusion were performed.

**Outcomes:**

Seven days after the treatments, the patient's symptoms resolved, the complete blood count, C-reactive protein, erythrocyte sedimentation rate were lowered. The size of the cyst was slightly reduced on 17 June compared to that before EBUS-TBNA. Although the surgical resection of the cyst was recommended, the patient declined. After extracted the two drainage tubes, the patient was discharged on June 22. The patient was followed up by telephone 6 months after discharge and he remained asymptomatic.

**Conclusions:**

EBUS-TBNA is a useful diagnostic and therapeutic tool for the management of mediastinal cysts. However, considering the possibility of serious complications, the clinical procedure should be carried out scrupulously with appropriate patient selection and strict aseptic principles.

## 1 Introduction

Convex Probe endobronchial ultrasound-guided transbronchial needle (EBUS-TBNA) has been conventionally used for the staging of lung cancer and sampling of mediastinal and hilar lymphadenopathy,^[1]^ and less commonly used in nonmalignant diseases.^[2–3]^ In particular, its application in mediastinal cystic lesions has been reported less frequently, mostly in case reports,^[4–8]^ with only a few cohort studies and systematic review of case reports.^[9–11]^ It shows that EBUS-TBNA is a reassuringly safe procedure with an overall complication rate of less than 2%,^[12–15]^ and serious adverse event rate of 0.14% to 0.16%.^[13,16]^ The most common complications are fever, infection (mediastinitis, pneumonia, mediastinal cyst infection, etc.), hemorrhage, and pneumothorax,^[12–14,16–17]^ unusual or rare complications include bronchoscope damage,^[12]^ needle breakage,^[18]^ pneumoperitoneum,^[19]^ haemotympanum,^[20]^ airway obstruction,^[21]^ subcutaneous emphysema,^[22]^ hemothorax,^[23]^ and pericardial abscess.^[24]^ The application of EBUS-TBNA in mediastinal cystic lesions is usually accompanied with a high incidence of infection.^[4,6,9,13,16,25]^ Herein, followed by a literatue review, we reported a patient with severe infections including pneumonia, pleurisy, mediastinitis and mediastinal cyst infection secondary to EBUS-TBNA, aiming to state the utility and complications of EBUS-TBNA in mediastinal cyst settings.

## 2 Case report

**Figure 1:**
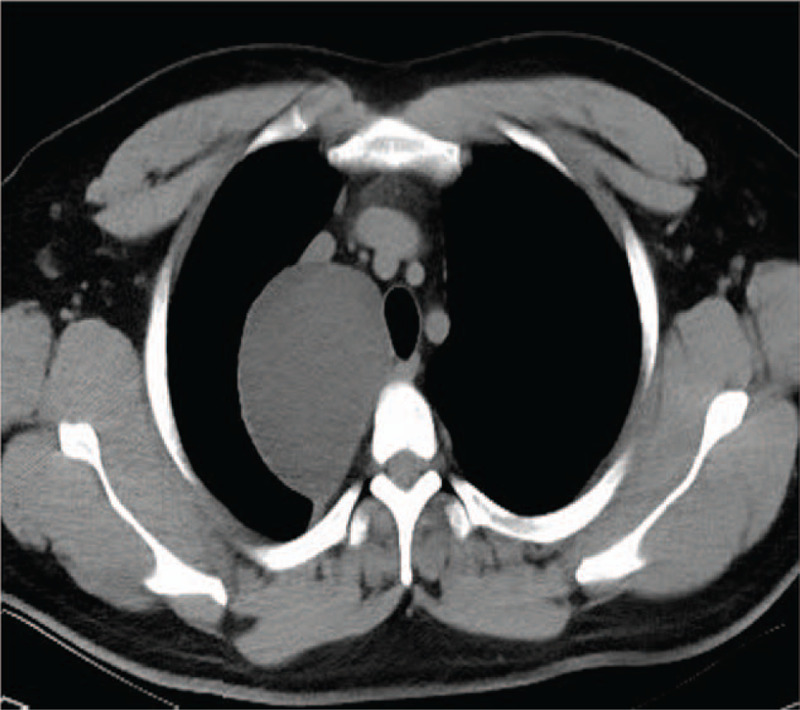
Chest computed tomography (CT) showed a thin wall cystic lesion with the size of 71 mm × 81 mm located in the posterior superior vena cava of the right superior mediastinum.

**Figure 2:**
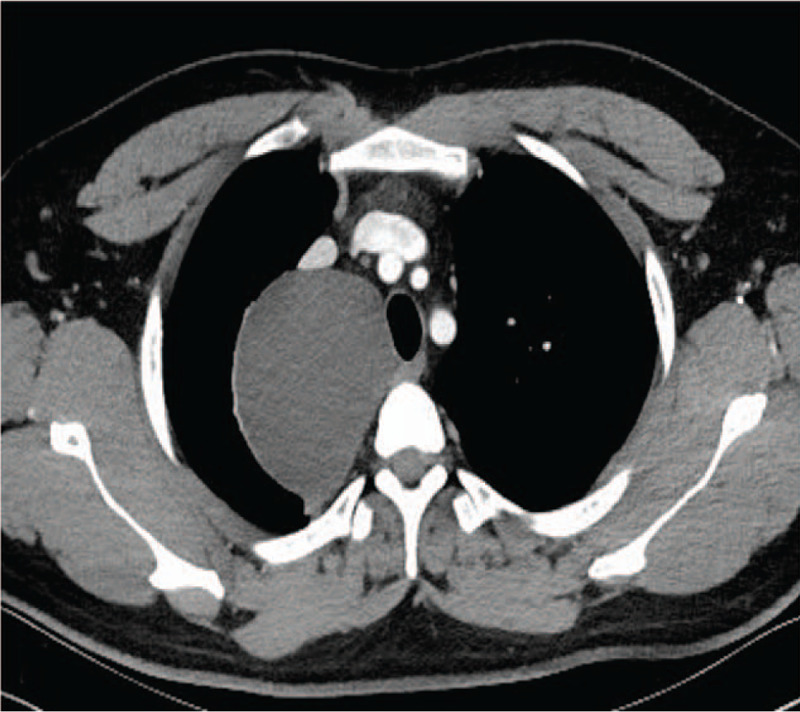
Contrast chest CT showed the enhancement of the wall of cystic lesion.

**Figure 3:**
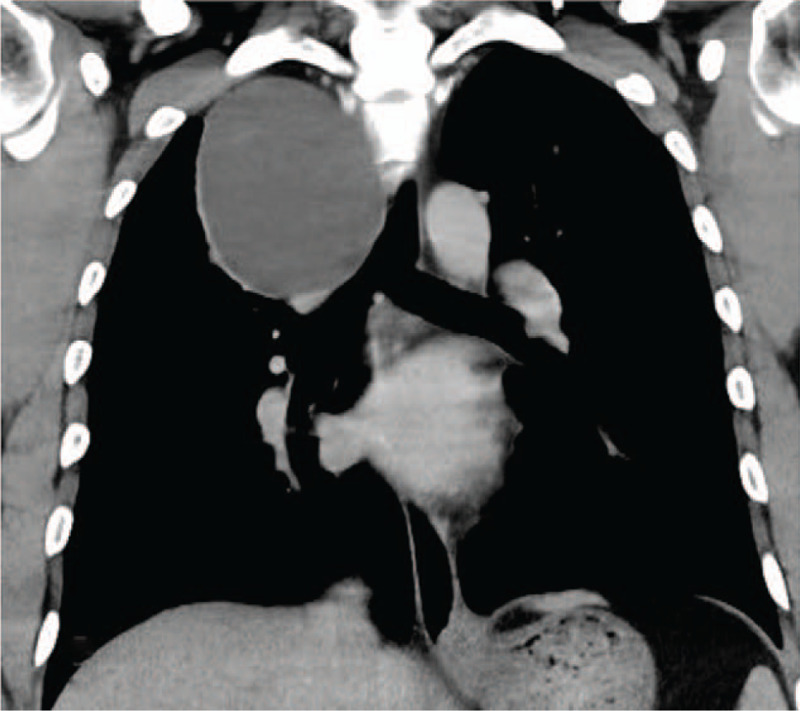
Coronal contrast CT.

**Figure 4:**
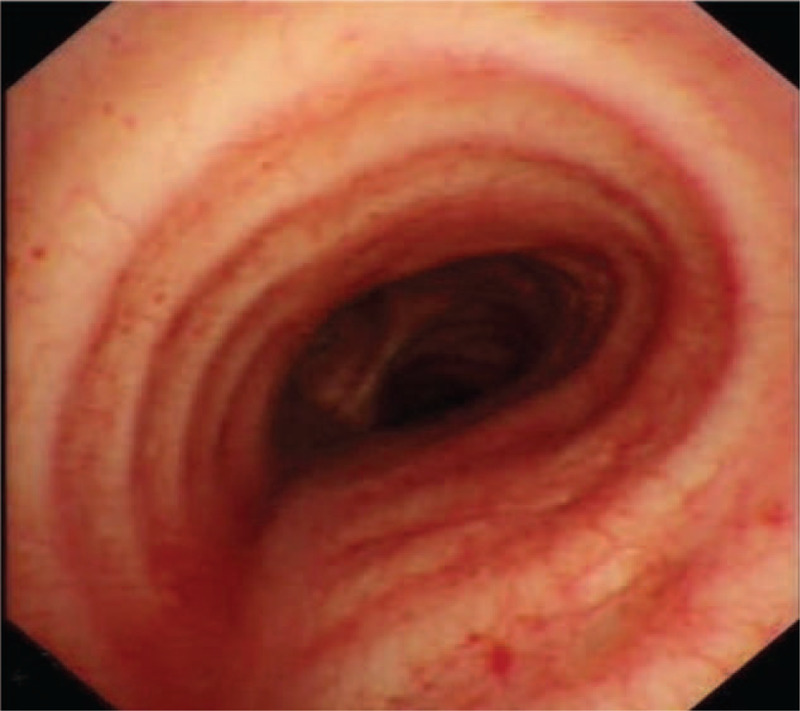
Bronchoscopy showed extrinsic compression without mucosal changes.

**Figure 5:**
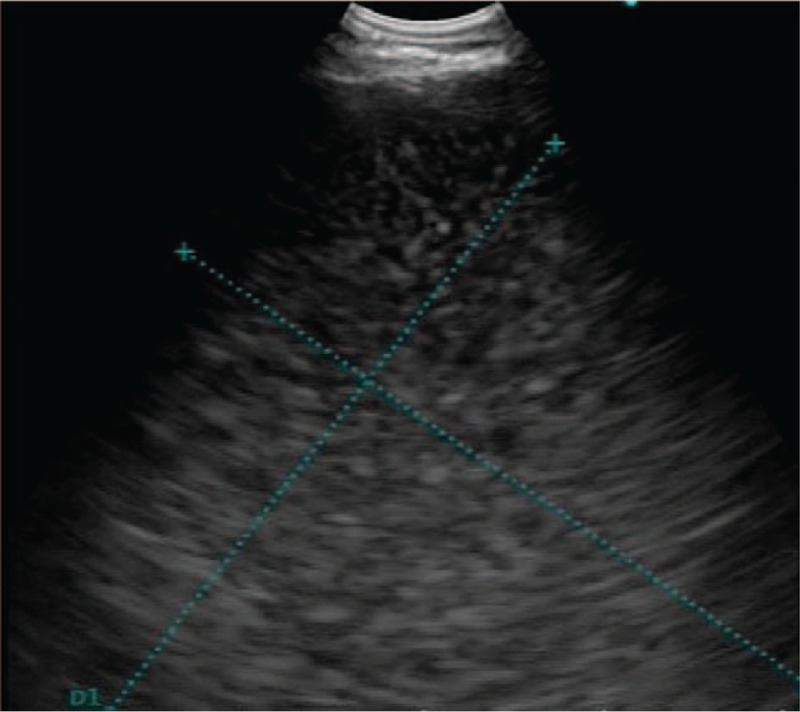
CP-EBUS revealed an well–circumscribed lesions of water density.

**Figure 6:**
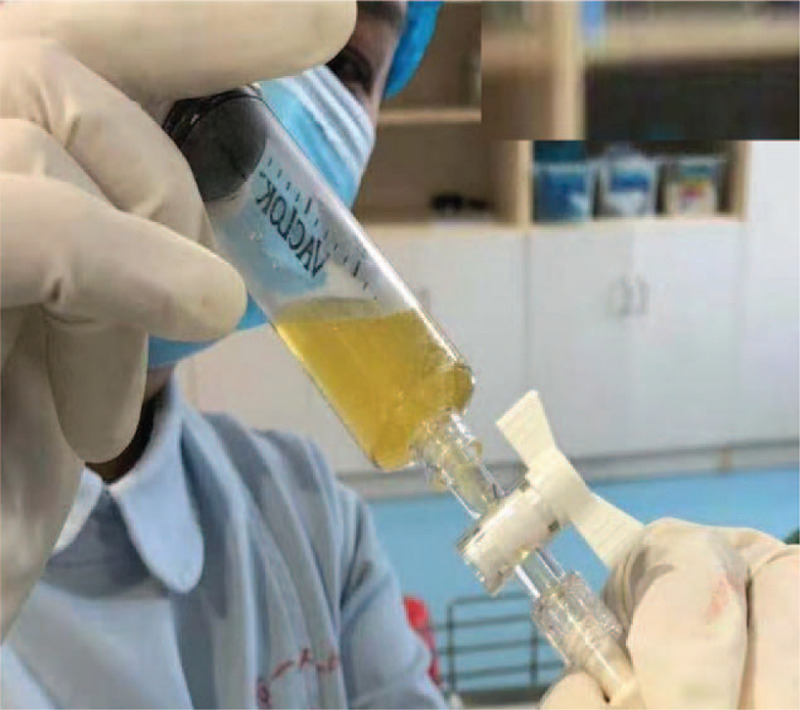
Clear colored yellow liquid was aspirated.

**Figure 7:**
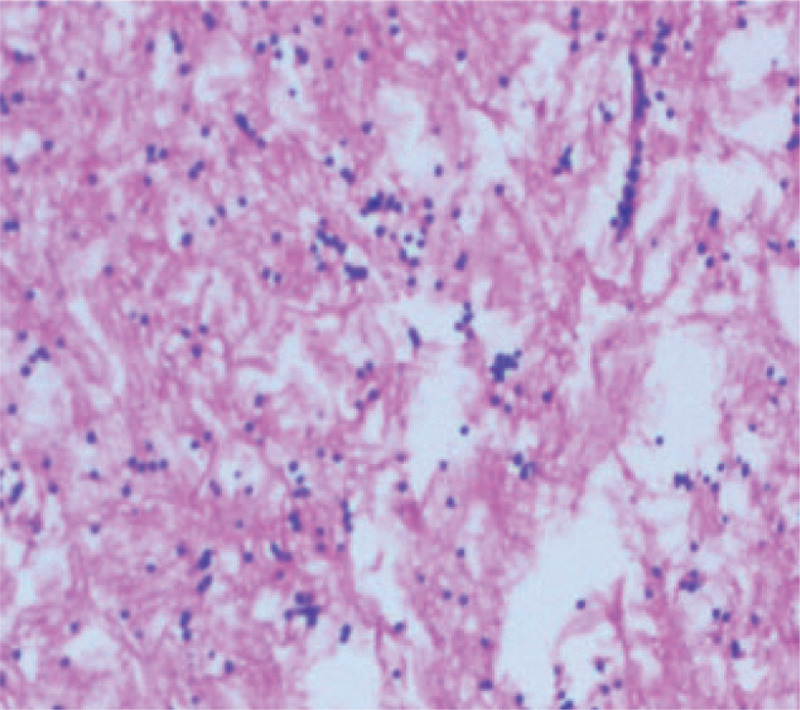
lymphocytes in the aspirated material (HEX20).

A 28-year-old male patient presented to the hospital with mediastinal cyst that was incidentally discovered by computed tomography (CT) on June 8,2020 (Figs. 1–3). He was asymptomatic and his lungs were clear on auscultation, and other physical examinations revealed no remarkable findings. Cyst size and the blood test were summarized in Table 1. Liver function, renal function and coagulation test or clotting profile were normal. Moxifloxacin was administered prophylactically. On the next day after admission, bronchoscopy were performed under conscious sedation (sufentanil and midazolam) as well as 2% lidocaine for topical anesthesia. The upper and middle trachea were presented with extrinsic compression without mucosal change (Fig. 4). Olympus EBUS scope (BF-UC260F; Olympus Ltd, Tokyo, Japan) was inserted orally using the 7.5 MHz convex probe. The cyst was identified as a round, anechoic structure. A dedicated 22-gauge needle (NA-201SX-4022, Olympus Ltd, Tokyo, Japan) was used to puncture and aspirate the lesion under real-time direct visual guidance (Fig. 5). The needle was inserted about 3 cm once, and serous fluid was aspirated with the total volume of 190 ml (Fig. 6). The aspirated fliud was sent for analysis, including Gram stain, bacterial culture, mycobacterial culture, and cytological analysis. Tests of aspirated fluid were summarized in Table 2. The carcino-embryonic antigen, mycobacterium tuberculosis DNA and all cultures, including acid fast bacilli, were negative. Cytology analysis of the aspirated fluid showed lots of lymphocytes and no malignant cells (Fig. 7). The diagnosis of lymphangioma was confirmed according to the CT and EBUS presentation, the nature of the aspirated fluid and the large number of mature lymphocytes within the cyst fluid.

**Table 1:** The cyst size and the blood test during hospitalization and outpatient follow-up.

	At admission (2020/6/8)	Onset of fever (2020/6/10)	2020/6/13	2020/6/17	2020/7/17	Reference ranges
Cyst size (mm)	71 X 81	74 X45	71 X 84	57 X 69	44 X 55	NA
WBC (X10^9^/L)	6.95	12.54	11.24	8.35	NA	3.5∼9.5
Neutrophil count (X10^9^/L)	4.7	10.28	8.18	5.87	NA	1.8∼6.3
Neutrophil percentage (%)	67	81.91	72.81	70.2	NA	40∼75
CRP (mg/L)	1	NA	131.6	48.2	NA	0∼8
ESR (mm/h)	2	NA	65	19	NA	0∼15
PCT (ng/ml)	<0.05	<0.05	<0.05	<0.05	NA	<0.05

**Table 2:** The analysis of cyst fluid and pleura effusion.

	EBUS-TBNA cyst fluid	Transcutaneous drainage cyst fluid	Pleura effusion
Total cell (X10^6^/L)	8137	62534	7600
WBC (X10^6^/L)	3137	40534	5760
LDH (U/L)	531.6	1545	295.7
Glucose (mmol/L)	4.27	0.2	4.51
ADA (U/L)	37.7	40.5	8.9
Total protein (g/L)	50.2	44.5	41.2

**Figure 8:**
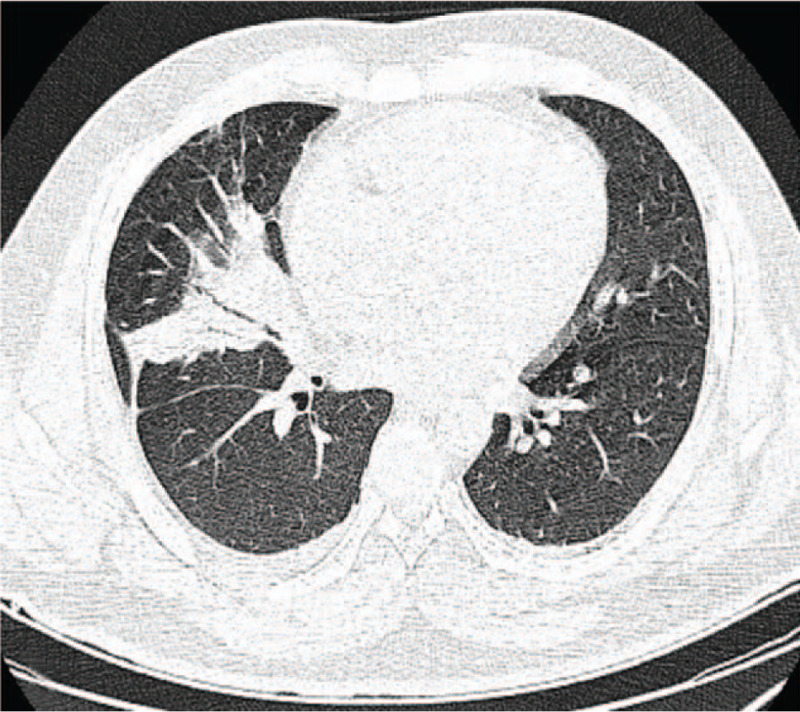
Chest CT on June 13 showed pneumonia.

**Figure 9:**
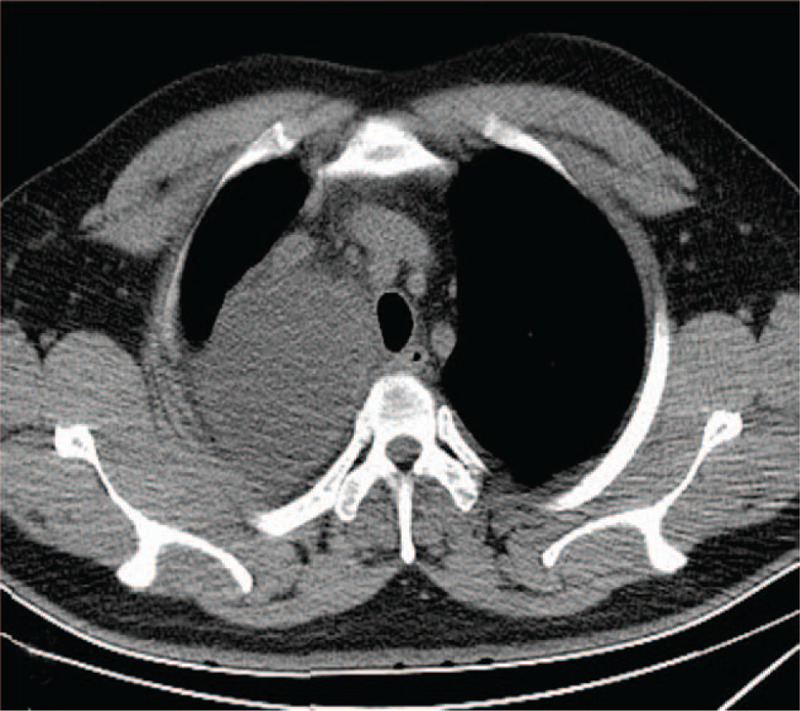
Chest CT on June 13 showed enlarged mediastinal cyst.

**Figure 10:**
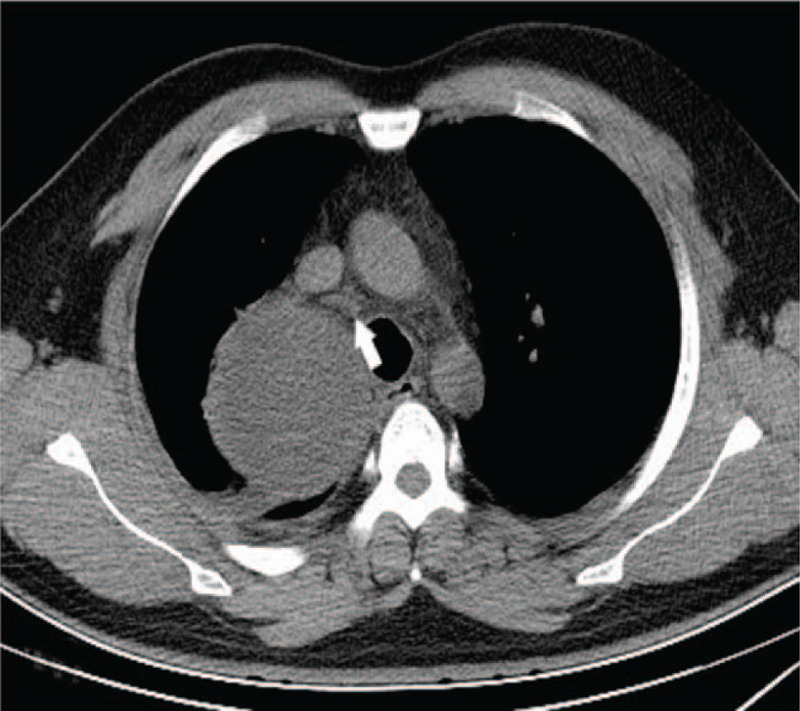
Chest CT on June 13 showed mediastinitis (arrows showing the higher mediastinal fat density).

**Figure 11:**
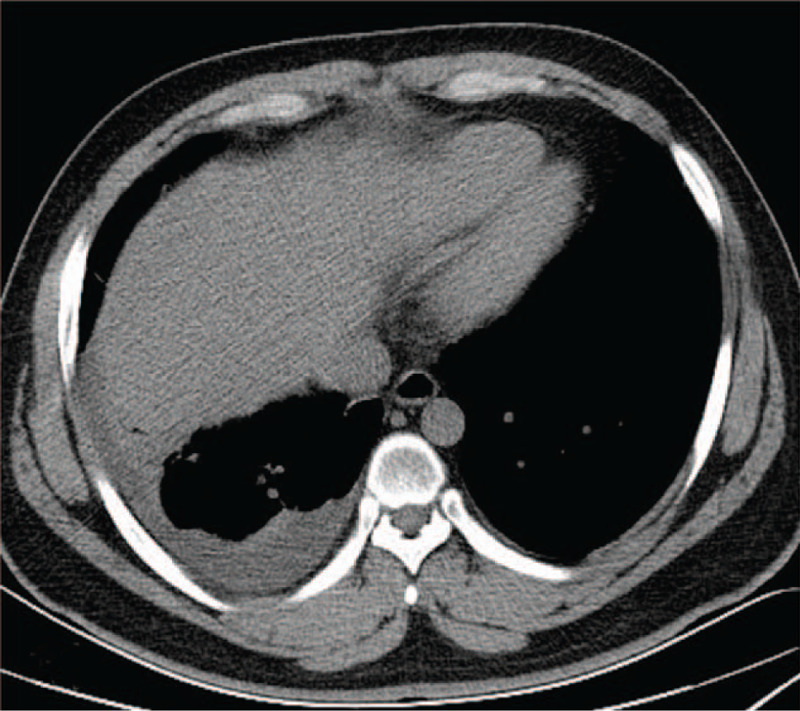
Chest CT on June 13 showed the right pleural effusion.

**Figure 12:**
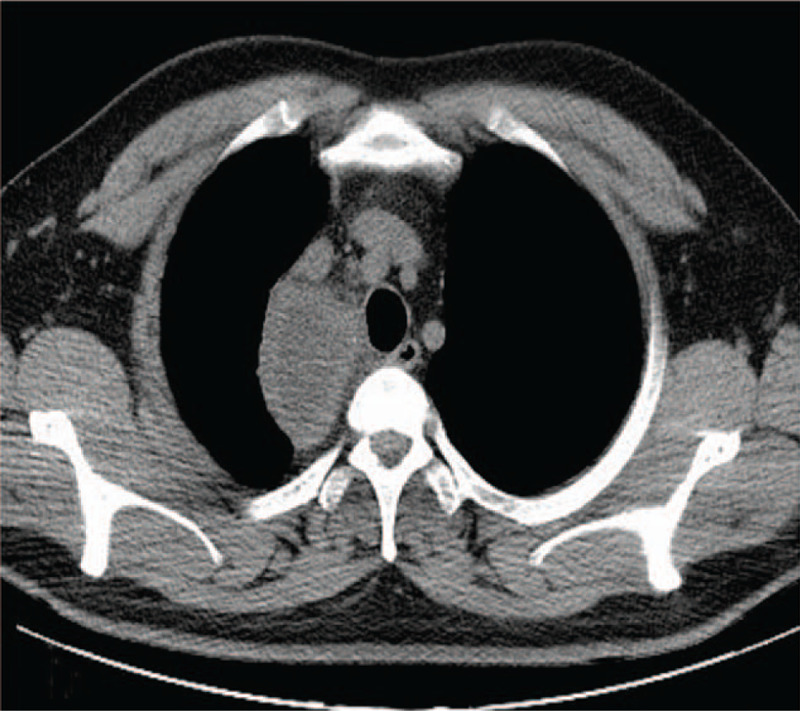
Chest CT on July 17 showed the cyst was smaller than the size of pre-EBUS-TBNA with a thicker wall.

Twenty-six hours after EBUS-TBNA, the patient complained of a fever with the highest temperature of 39°C, accompanied by a right-side chest pain, no obvious symptoms of chill, cough, expectoration of sputum and shortness of breath. Repeated chest CT imaging showed that the cyst was smaller than that before EBUS-TBNA, with a size of 74 mm × 45 mm, chest CT also showed pneumonia, pleural effusion of the right side and increased mediastinal density (Figs. 8–11). The results of infection indicators were shown in Table 1. Considering secondary pneumonia, pleurisy, mediastinitis and cyst infection, the patient was treated with Teicoplanin+Imipenem/cilastatin, and transcutaneous catheterization drainage of mediastinal cyst and right pleural effusion with ultrasound guided were performed on June 13, 2020. The examination results were shown in Table 2, cultures from mediastinal cyst and right pleural effusion were both negative. Seven days after the treatment, the patient's symptoms resolved, the complete blood count, C-reactive protein, erythrocyte sedimentation rate were lowered. The size of the cyst was slightly reduced on 17 June compared to that before EBUS-TBNA. Although the surgical resection of mediastinum cyst was recommended, the patient declined our proposal. The two drainage tubes were extracted and the patient was discharged home on June 22. On 17 July, the cyst size reduced to 44 mm × 55 mm asymptomatic (Fig. 12). The patient was followed up by telephone 6 months after discharge and he remained asymptomatic.

## 3 Discussion

Mediastinal cystic lesions account for 12% to 18% of primary mediastinal tumors, with bronchial cysts being the most common (40%), followed by pericardial cysts (35%), enterogenous cyst (10%), and unclassified (14%).^[26]^ About two-thirds of patients with mediastinal cyst remain asymptomatic. However, some patients present with symptoms due to compression of adjacent organs, cyst infection, etc.^[21]^ The common symptoms are chest pain, dyspnea, hemoptysis or cough and expectoration due to recurrent cyst infections.^[26]^ Chest CT or magnetic resonance imaging (MRI) examinations are clinically important for the diagnosis of mediastinal cysts. Typical mediastinal cysts are presented as liquid density (Hounsfield Unit<20) on CT images, mostly round or lobulated, with clear boundaries and no enhancement in contrast CT. However, some infected cysts, proteinaceous, or combined with hemorrhage or calcification have atypical CT manefestations, making them indistinguishable from neoplasms. The accuracy of CT in the diagnosis of mediastinal cysts was reported by Aravena et al to be only 53.8%,^[11]^ about 43% of cysts could be misdiagnosed as solid mediastinal tumors.^[11]^ An MRI is also helpful in the diagnosis of mediastinal cysts, and serous fluid has a low T1-weighed signal and high T2-weighted signal images.^[27]^ For some proteinaceous, hemorrhagic or infected cysts, MRI images are atypical and may show higher T1-weighed signal images. EBUS has unique advantages in diagnosing mediastinal cysts. It may display anechoic (black), hypoechoic (darker), isoechoic (grey), and visible separation in the cyst. The Doppler patterns can be used to differentiate vascular structures.^[28]^ For some cysts with features of solid tumors on CT images due to the presence of proteinaceous content, the fluid-fluid level could be seen on EBUS, which is a more typical feature and can be used to differentiate cysts with features of solid tumors and neoplastic lesions.^[29]^ The fluid-fluid level of noninfected cyst is probably due to the dependent layering of proteinaceous material within the cyst.^[29]^ However, it is difficult to differentiate cysts with tumor necrosis.^[9]^

For cysts that are difficult to diagnose on imaging (including CT, MRI, EBUS), surgical excision of the cyst is of definite significance for the diagnosis and identification of the lesions origin. However, for patients who are unfit or reluctant for surgery, transcutaneous, transesophageal or transbronchial aspiration of the cystic fluid and analysis of its cellular composition can help to identify the origin of the cyst.^[11]^ The origin of the cyst is determined based on its cell components on the cytology specimen: bronchogenic cyst if bronchial epithelium were present, thoracic duct cyst or lymphangioma if lymphocytes were present, pleural or pericardial cyst if mesothelial cells were present, hydatid cyst if hydatid scolices were present, tuberculosis if acid-fast bacilli were present and cystic neoplasm if malignant cells were present.^[9,11]^ However, the sensitivity of cyst composition analysis to determine the origin of cysts is undesirable, a study by Aravena C^[11]^ showed that only 27% of cysts were able to identify the origin by an analysis of the cysts composition. Nevertheless, from a therapeutic point of view, this does not affect the application of EBUS-TBNA in mediastinal cystic lesions, as patients usually presented with symptoms of compression of adjacent organs by oversized cysts or infection, independent of the cyst origin.^[11]^

Due to the rare nature of mediastinal cysts, there is still little evidence to guide management, and clinical recommendations are mostly derived from case reports.^[9]^ For symptomatic mediastinal cysts, it is agreed that surgical removal of the cyst is a more reasonable option, while the choice of treatment for asymptomatic patients still remains controversial.^[11]^ Other than surgery, aspiration of the cyst is still an option, and it can be performed transcutaneous, transesophageal or transbronchial according to the location. Transbronchial aspiration seems to be a feasible option, as most of the mediastinal cysts are located near the trachea or bronchi.^[9]^ With the widespread use of EBUS-TBNA in clinical practice, the position of the needle tip can be monitered under ultrasound guidance and the depth of puncture can be adjusted, which is conducive to adequate drainage of the cystic fluid, especially for multifocal cysts.^[4]^ Zhong and colleagues reported that EBUS-TBNA has significant therapeutic effects on mediastinal lymphangioma without serious complications. The disease was stable over a period of 9 months to 2 years.^[10]^ However, another study reported an efficacy of no more than 46% after aspiration with EBUS-TBNA, with only 5.5% of patients achieving complete remission.^[11]^ The use of EBUS-TBNA has related complications such as secondary infection and hemorrhage, especially for cystic lesions, which seems to have a greater potential to develop cystic infection, lung abscess, pleural infection or mediastinal infection than solid lesions of the mediastinum.^[4,6,9,13,16,25]^ The reported infectious pathogens include Streptococcus,^[5–6,16–17,24–25,30–31]^ Coryneform bacteria and Prevotella melaninogenica,^[5]^ Staphylococcus,^[4,24]^ Mycobacterium tuberculosis,^[21]^ Haemophilus Influenza,^[32]^ Eikenella corrodens,^[24,33]^ Klebsiella pneumonia, Actinomyces odontolyticus, Gemella morbillorum, Prevotella buccae, Actinomyces,^[30]^ among which the most of the reported bacteria are oral flora. During the procedure of EBUS-BNA, the bronchoscope and needle may be contaminated when passing through the oropharynx, thus carrying bacteria of the oral flora into the cyst. Mediastinal cysts, however, have no vascular component and have a compromised ability to eliminate bacteria, and therefore are predisposed to secondary infections. Other sources of contamination include needle contamination by slides during specimen preparations, inadequate instrument sterilization.^[34]^

Hence, on the basis of our experience and literature review, we suggest judicious use of EBUS-TBNA as a therapeutic tool for mediastinal cystic lesions as it might be associated with increased risk for infection. In conclusion, EBUS-TBNA is a useful diagnostic and therapeutic tool for the management of mediastinal cysts, including lymphangioma. However, considering the possibility of serious complications, the clinical procedure should be carried out scrupulously with appropriate patient selection and strict aseptic principles.

## Acknowledgments

The authors express their gratitude to Dr E.K. Makoni in Parirenyatwa group of Hospital in Zimbabwe and Dr Jie Shu in Xiangya Hospital, Central South University in China for their advices on language editing.

## Author contributions

**Funding acquisition:** Zhi-Guang Liu.

**Investigation:** Wei-Dong Zhang, Huai-Qiu Wu, Zhi-Guang Liu.

**Resources:** Yong-Xue Wang.

**Writing – original draft:** Wei Liu.

**Writing – review and editing:** Wei Liu.
